# Structural and cross-cultural validity of the Afrikaans for the Western Cape Disabilities of the Arm, Shoulder and Hand (DASH) questionnaire

**DOI:** 10.1186/s41687-022-00536-w

**Published:** 2023-01-11

**Authors:** Susan de Klerk, Christina Jerosch-Herold, Helen Buchanan, Lana van Niekerk

**Affiliations:** 1 Division of Occupational Therapy, Department of Health and Rehabilitation Science, Faculty of Medicine and Health Science, Stellenbosch University, Cape Town, South Africa; 2 School of Health Sciences, University of East Anglia, Norwich, UK; 3 Division of Occupational Therapy, Department of Health and Rehabilitation Sciences, Faculty of Health Sciences, University of Cape Town, Cape Town, South Africa

**Keywords:** Internal consistency, Measurement invariance, Multiple group confirmatory factor analysis, South Africa, Upper limb impairment

## Abstract

**Background:**

The Disabilities of the Arm, Shoulder and Hand (DASH) questionnaire has been translated and cross-culturally adapted to Afrikaans for the Western Cape, within the public health service context of South Africa. The aim of this study was to evaluate structural validity, internal consistency, and cross-cultural validity/measurement invariance of this new translation to increase applicability and clinical utility in a public health service context.

**Methods:**

During this cross-sectional study, exploratory factor analysis (EFA) was conducted with parallel analysis and oblimin rotation. Confirmatory factor analysis (CFA) and multiple group confirmatory factor analysis (MGCFA) to assess cross-cultural validity/measurement invariance, was employed to test model fit with *X*^2^ goodness-of-fit statistic, root mean square error of approximation (RMSEA), standardized root mean square residual (SRMR) and comparative fit index (CFI). Internal consistency was calculated using Cronbach’s alpha.

**Results:**

109 women and 110 men (n = 219) completed the Afrikaans for the Western Cape and the South African English DASH questionnaire, used during the analysis. Unidimensionality of the Afrikaans for the Western Cape DASH questionnaire was not supported in the 218 questionnaires eligible for inclusion in the analysis [*X*^2^ (df) = 1799.10 (405); *p* value = < 0.01; RMSEA (90% CI) = 0.126 (0.120–0.132); SRMR = 0.09 and CFI = 0.984]. EFA revealed a two-factor structure with Eigenvalues exceeding one explaining 55% and 7% of the variance. The two-factor structure of the Afrikaans for the Western Cape DASH questionnaire was supported during CFA. Cronbach’s alpha revealed good internal consistency of both factors [factor 1 = 0.97 (0.96, 0.97) and factor 2 = 0.92 (0.90, 0.94)]. MGCFA conducted between 218 Afrikaans for the Western Cape DASH and 219 South African English DASH questionnaires (N = 437) revealed that the data supports configural, metric and scalar invariance models during initial model fit assessment. Subsequent hypotheses testing comparing the nested models revealed that scalar invariance holds.

**Conclusion:**

The Afrikaans for the Western Cape DASH questionnaire revealed a two-factor structure with good internal consistency across the two factors and demonstrated measurement invariance with the South African English DASH questionnaire.

**Supplementary Information:**

The online version contains supplementary material available at 10.1186/s41687-022-00536-w.

## Background

South Africa faces a high burden of upper limb conditions and injuries. Best practice for intervention toward addressing patient needs includes the utilisation of valid and reliable outcome measures. One such region-specific patient reported outcome measure (PROM) is the Disabilities of the Arm, Shoulder and Hand (DASH) questionnaire [1]. The DASH questionnaire measures symptoms and physical function in patients with upper limb conditions [1] and has been extensively studied across the globe. As it was developed in English (Canada), translation and cross-cultural adaptation must be conducted, followed by the assessment of psychometric properties of the new language version [2, 3]. South Africa continues to be the country with the highest GINI coefficient (a measure of income inequality) in the world (0.63), highlighting inequality in all spheres of life. This inequality extends to the health sector, with 84% of the population utilising the public health sector which is overburdened and poorly resourced [4, 5]. Health services are designed to address issues of inequality [5]. The availability of PROMs, validated in the public health service context would contribute towards that goal as accurate self-report on aspects of activity and participation or quality of life could change how healthcare is delivered [6] and potentially improve health outcomes, thus addressing the social determinants of health [7, 8]. Finally, the availability of the DASH questionnaire in a low-resource health care setting like South Africa may facilitate future work to increase the availability and uptake of similar PROMS in other low resource health settings across the globe.

Self-report measures need to be available in the patients’ preferred language. As a result of the multilingualism within South Africa (11 national languages and many others without status) and among the 84% of South Africans that access the public health sector, many translations of PROMs should exist. English (20.3%), Afrikaans (49.7%) and isiXhosa (24.7%) are the three most predominantly spoken languages in the Western Cape of South Africa. The variation that exists within the Afrikaans language (previously described [9]) served as motivation to translate and cross-culturally adapt the DASH questionnaire into Afrikaans for the Western Cape, within the public health context of the Western Cape of South Africa and is reported on elsewhere [10, 11]. Validity of this new language version and equivalence between language versions within this context would allow for accurate self-report and for comparisons between patient scores (from different languages and cultural backgrounds) within the same clinical setting, addressing the call to demonstrate outcomes of intervention [12].

The objectives of this research were therefore to evaluate (1) the structural validity and internal consistency of the newly translated and cross-culturally adapted Afrikaans for the Western Cape DASH questionnaire and (2) the cross-cultural validity (measurement invariance) between the Afrikaans for the Western Cape and the South African English DASH questionnaires. This quantitative evaluation of the construct validity of the South African English DASH questionnaire was nested within a larger overarching study as no prior evidence existed [13]; the results are presented in Additional file 1.

## Methods

### Research context: population and setting

Adult patients (older than 18 years of age) with injuries and conditions of the upper limb, attending an outpatient upper limb clinic appointment between September and December 2021 within the Department of Orthopaedic Surgery at Tygerberg Academic Hospital (Cape Town, South Africa) were recruited to participate in this cross-sectional study. An inclusion criterion was the ability to read both English and Afrikaans towards completion of the questionnaires. Consent was sought prior to participation and patients were given fruit juice, a snack, and the pen they used to complete the questionnaire as a token of appreciation for their participation.

### Data collection and instrumentation

The 30-item DASH questionnaire comprises 21 items on physical function, six items on symptoms and three psychosocial items [2]. For each item the patient selects a response category from a five-point Likert scale which represents severity or level of difficulty. No more than three missing responses are allowed to calculate the overall DASH score. The total score is calculated as follows: (sum of responses/number of responses) − 1 × 25 with scores closer to 100 indicating greater disability. The Afrikaans for the Western Cape DASH (for which the translation and cross-cultural adaptation and content validity is described elsewhere [10, 11]) and the South African English DASH (available from the IWH website: https://dash.iwh.on.ca/available-translations; with evidence of construct validity available in Additional file 1) were used during data collection. Patients were requested to complete both language versions.

### Structural validity, internal consistency and cross-cultural validity

The internal structure of a measure is reflected in the measurement properties, namely structural validity, internal consistency and cross-cultural validity (measurement invariance) [14]. Internal structure in its simplest form refers to an understanding of how the different items on a measure relate to each other [14].

The assessment of structural validity is implied in measures based on a reflective model, towards establishing the dimensionality of the instrument [14, 15]. Unidimensional instruments are summed into a single score as, once established that the measure is, in fact, unidimensional, it is understood that all the items reflect a single construct. The Consensus-based Standards for Selection of Health Measurement Instruments (COSMIN) definition of structural validity was adopted in the current study and refers to the extent to which the scores obtained on an instrument are an adequate reflection of the dimensionality of the construct [15]. In other words, they address the question of whether items load on a single factor (unidimensional) or several factors (multidimensional). This is done through either confirmatory or exploratory factor analysis. confirmatory factor analysis (CFA) is preferred when there is existing evidence or a previous hypothesis about the number of factors that exist for the measure [16–23]. CFA was utilised in the present study to evaluate a one-factor structure in the Afrikaans for the Western Cape DASH questionnaire. If unidimensionality is not confirmed, exploratory factor analysis (EFA) can be employed to assess the number of factors that exist in the measure as EFA is a data-driven method, that allows the exploration of dimensionality [15]. Internal consistency is evaluated by item-total correlation in unidimensional scales (or subscales) and expressed as Cronbach’s alpha [14, 15].

Cross-cultural validity needs to be assessed in measures that have been translated and cross-culturally adapted for use in populations that are different from the one that the measure was originally developed for [14]. Cross-cultural validity in the current study was concerned with measurement invariance (MI) across language versions of the DASH questionnaire, specifically the newly translated Afrikaans for the Western Cape and the South African English DASH questionnaire. MI, also referred to as measurement equivalence, implies that an instrument will behave in the same way in the population under investigation, irrespective of the language used [14, 15, 24]. This is an important consideration in the culturally and occupationally diverse population with upper limb conditions and injuries within South Africa. Therefore, cross-cultural validity (MI) was assessed through multiple group confirmatory factor analysis (MGCFA) for the variable “language” (as all other variables were the same) to ensure equivalence between language versions [15, 24].

### Statistical analysis

The sample size was determined based on COSMIN guidelines [25] which suggest an adequate sample size for conducting CFA and MGCFA is seven times the number of items in the PROM and ≥ 100. In the case of the DASH questionnaire, 210 participants are required. All analyses were performed using R’s package lavaan [26]. CFA and MGCFA appropriate fit indices were considered. In addition to *X*^*2*^ goodness-of-fit statistic (known to be sensitive to sample size) the following fit indices were used to evaluate model fit: root mean square error of approximation (RMSEA) close to or less than 0.06, standardized root mean square residual (SRMR) close to or less than 0.08 and comparative fit index (CFI) close to or higher than 0.95 were considered adequate. EFA was conducted by exploring the number of factors through parallel analysis, a robust factor retention method [27, 28]. Internal consistency was calculated for each scale (subscale) included in the analysis with Cronbach’s Alpha. MI was assessed with MGCFA; the three MI steps that were considered were configural, metric (also known as weak factorial) and scalar (also known as strong factorial) invariance [29, 30]. These three steps addressing the questions outlined by De Vet et al. towards providing evidence of cross-cultural validity [31] were: (1) are the same factors identified in both language versions; (2) are the factor loadings the same in both language versions; and (3) are the mean values the same between language versions? Model fit indices were applied as outlined above. In addition, null hypotheses (that the invariance models fit the data equally) were tested for metric invariance (the fit between configural and metric models) and scalar invariance (the fit between metric and scalar invariance models) to ascertain which best supports invariance.

## Results

### Descriptive statistics

A cohort of 219 (109 women and 110 men) patients, bilingual in Afrikaans and English, were recruited and agreed to participate in the study. The mean age of the participants was 40.6 years (SD = 13.9). The frequency of upper limb condition or injury can be seen in Table 1, with the hand being most frequently affected in the sample (n = 93, 42.5%) followed by the forearm and wrist (n = 84, 38.4%).

**Table 1:** Frequency of upper limb injury/condition (n = 219)

Injury or condition affecting the shoulder, upper arm, elbow, forearm and wrist or hand	n (%)
Shoulder	16 (7.3)
Upper arm	22 (10.0)
Elbow	4 (1.8)
Forearm and wrist	84 (38.4)
Hand	93 (42.5)

Educational level, employment status, duration of symptoms in weeks and time taken to complete the questionnaires can be seen in Table 2. Participants completed both the Afrikaans for the Western Cape and the South African English DASH questionnaire on the same occasion after being presented in random order. In the Afrikaans for the Western Cape DASH Questionnaire the highest percentage of missing responses was for item 3 [Turning a key (4.1%)] and in the South African English DASH Questionnaire for item 21 [Sexual Activity (4.6%)]. Total DASH scores could be calculated for 218 of the Afrikaans for the Western Cape and 219 of the English DASH Questionnaires.

**Table 2:** Demographic information (n = 219)

Education level and employment status	n (%)
*Educational level*	
Primary school	12 (5.5%)
High school	187 (85.4%) of whom 73 (33.3%) graduated high school
Tertiary (university/college)	18 (8.2%)
Not recorded	2 (0.9%)
Total	219 (100%)
*Employment status*	
Employed	110 (50.2%)
Unemployed	101 (46.1%)
Retired	6 (2.7%)
Not recorded	2 (0.9%)
Total	219 (100%)

Duration of symptoms/time since injury	Median (min–max)
	8 (1–1248) weeks

Time taken to complete DASH	Mean (SD)
Afrikaans for the Western Cape	8.0 (2.8) min
South African English	7.8 (2.4) min

### Structural validity and internal consistency

**Fig. 1:**
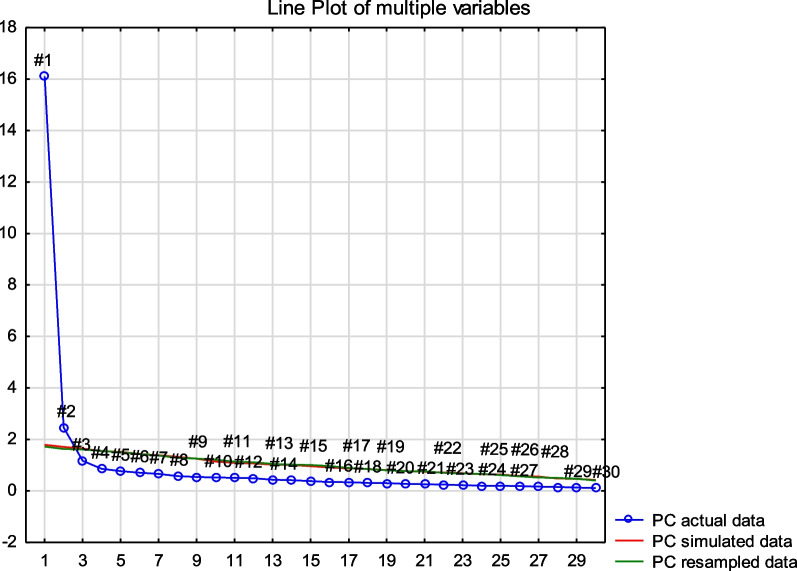
Scree plot parallel analysis (Afrikaans for the Western Cape)

The CFA conducted on the Afrikaans for the Western Cape DASH questionnaire did not support a one-factor structure of the instrument as the fit indices were as follows: *X*^2^ (*df*) = 1799.10 (405); *p* value =  < 0.01; RMSEA (90% CI) = 0.126 (0.120–0.132); SRMR = 0.09 and CFI = 0.984 and as a result did not meet the requirements for good model fit. We then proceeded to conduct EFA to explore the number of factors within the measure. The scree plot with parallel analysis (Fig. 1) of the observational data (n = 218) and the random data set without structure (with the same number of variables and observations) tailed off after two factors in the observational data.

It can also be seen on the scree plot that the two factors identified in the observational data lie above the line created by the random data, a rule of thumb for retaining the two factors [28]. The two factors with Eigenvalues exceeding one explained 55% and 7% of the variance to the cumulative value of 55% and 62%. The Kaiser–Meyer–Olkin (KMO) measure of sampling adequacy value was 0.96 (above the recommended value of 0.6[32]) and statistical significance was reached with Bartlett's test of Sphericity[33]: *X*^2^ = 5407.22, *df* = 435, *p* =  < 0.01. This supports the factorability of the data. Table 3 outlines the pattern matrix from EFA following Oblimin rotation. Items 1 to 21 loaded on one factor and items 22, 24–30 on the second factor. Item 23 (Limitations in work and daily activities) just achieved commonality with factor two with a value of 0.5 (values > 0.5 indicate good fit). We considered the individual items in each factor and determined that factor 1 (items 1–21) is reflective of physical function and factor 2 (items 22, 24–30) is reflective of biopsychosocial symptoms and as such it makes clinical sense for item 23 to contribute to factor 2. Internal consistency for each factor (unidimensional subscale) was assessed through Cronbach’s Alpha and results are presented in Table 3.

**Table 3:** Pattern matrix from EFA following oblimin rotation

DASH items (short description)	Factor 1 Physical Function	Factor 2 Biopsychosocial symptoms
1 (Opening a jar)	− 0.7	
2 (Writing)	− 0.51	
3 (Turning a key)	− 0.65	
4 (Meal preparation)	− 0.84	
5 (Pushing open a door)	− 0.58	
6 (Placing object overhead)	− 0.71	
7 (Heavy household chores)	− 0.81	
8 (Gardening)	− 0.85	
9 (Making a bed)	− 0.74	
10 (Carrying a bag)	− 0.66	
11 (Carrying heavy object)	− 0.74	
12 (Changing light bulb overhead)	− 0.76	
13 (Wash and blow-dry hair)	− 0.84	
14 (Back washing)	− 0.89	
15 (Donning pullover jersey)	− 0.8	
16 (Use knife to cut food)	− 0.82	
17 (Recreational activities—little force)	− 0.8	
18 (Recreational activities—some force)	− 0.9	
19 (Recreational activities—moving arm freely)	− 0.9	
20 (Transportation)	− 0.66	
21 (Sexual activities)	− 0.58	
22 (Interference with social activities)		− 0.59
23 (Interference with work)	− **0.32**	− **0.5**
24 (Pain)		− 0.93
25 (Pain during activity)		− 0.82
26 (Pins and needles)		− 0.88
27 (Weakness)		− 0.81
28 (Stiffness)		− 0.69
29 (Sleeping)		− 0.74
30 (Psychosocial function)		− 0.6
Eigenvalue	16.58	2.08
Percentage variance	55%	7%
Cumulative percentage	55%	62%
Cronbach’s alpha (95% CI)	0.97 (0.96, 0.97)	0.92 (0.90, 0.94)

CFA of the two-factor model derived from the EFA revealed that the two-factor structure of the Afrikaans for the Western Cape DASH was supported by the fit indices as *X*^*2 (df)*^ = 668.25 (404), *p* < 0.01, RMSEA (90% CI) = 0.055 (0.047–0.063), SRMR = 0.06 and CFI = 0.997.

Factor loadings for this two-factor model were high and ranged from 0.597 to 0.896 supporting the fit indices in confirming two factors (physical function and biopsychosocial symptoms) of the Afrikaans for the Western Cape DASH questionnaire.

### Cross-cultural validity (measurement invariance)

MGCFA was used to assess the measurement invariance for “language” between the Afrikaans for the Western Cape (n = 218) and the South African English DASH questionnaire (n = 219). As outlined above, configural, metric and scalar invariance models were tested by combining the two DASH questionnaires in the analysis (N = 437) and all three models indicated good fit during this initial assessment (Table 4).

**Table 4:** Multiple group confirmatory factor analysis of model fit—statistics

MGCFA	N	*X*^2* (df)*^	*p*	RMSEA (90% CI) [Fit supported if RMSEA close or ˂ 5]	SRMR [Fit supported if SRMR close or ˂ 0.08]	CFI [Fit supported if CFI close or to or ˃ 0.95]
M1: configural invariance	437	1428.66 (808)	< 0.001	0.058 (0.054–0.064)	0.05	0.997
M2: metric invariance	437	1544.00 (836)	< 0.001	0.062 (0.058–0.067)	0.06	0.996
M3: Scalar invariance	437	1490.05 (924)	< 0.001	0.053 (0.048–0.058)	0.05	0.992

M1 = Model 1, M2 = Model 2; M3 = Model 3; MGCFA = Multiple Group Confirmatory Factor Analysis; *X*^*2*^ = Chi Square*; df* = degrees of freedom; RMSEA = Root mean square error of approximation; CI = Confidence Interval; SRMR = Standardized root mean square residual; CFI = Comparative fit Index

Note: N = 437 (Afrikaans n = 218, English n = 219)

Even though all three models (configural, metric and scalar invariance) fit the data, comparing nested models is an important next step to confirm which one fits best [29]. The nested model comparison output is the difference in *X*^*2*^, the corresponding degrees of freedom and test of significance (*p* > 0.05). Table 5 outlines the results of comparing the models. The M1: configural and M2: metric models were compared hypothesising that both models fit the data equally (evaluating if metric invariance is supported). From the significant *p* value (less than 0.001), we infer that they do not fit the data equally and conclude that metric invariance is not supported.

**Table 5:** Nested model comparison (hypotheses testing)

Invariance model	*df*	*X*^2^	*X*^*2*^diff	*df*.diff	*p* value	Comment
Configural	808	1428,662				
Metric	836	1544,002	115,34	28	< 0.001	Metric invariance not supported
Scalar	924	1490,047	− 53,955	88	1	Scalar invariance supported

*df* = degrees of freedom; *X*^2^ = Chi Square; diff = difference

**Fig. 2:**
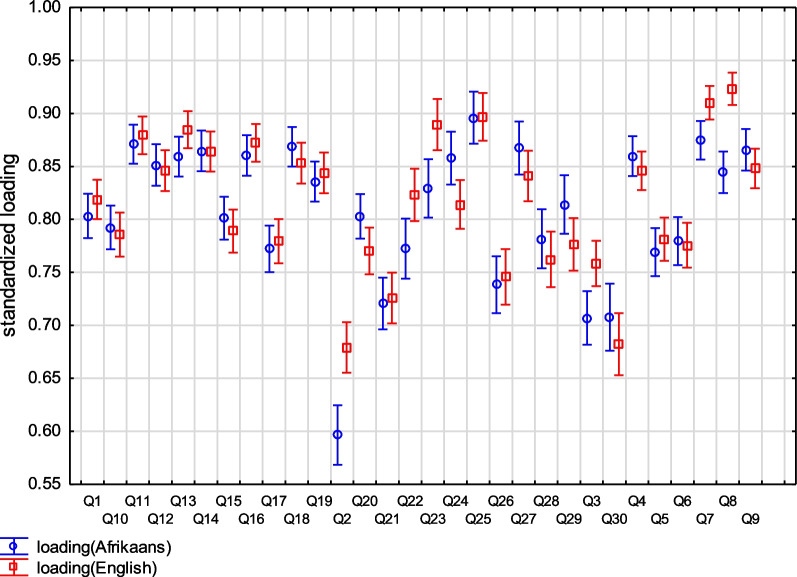
Range plot of standardise loading between Afrikaans for the Western Cape and English DASH questionnaires

The implication at this stage is that the loading (of items per factor) is not the same between language versions, as illustrated in the range plot (Fig. 2). The higher the loading (closer to 1) the more it contributes to the factor in question in the respective language versions. All factor loadings are high (0.597 and above) and closely plotted, with the biggest discrepancies being items 2 (writing), 3 (turning a key), 7 (doing heavy household chores), 8 (garden or do yard work) and 22 (interference with social activities). In both language versions these items load on the same factor but more so in the English version (item 2 = 0.679; item 3 = 0.758; item 7 = 0.91; item 8 = 0.923 and item 22 = 0.823) as compared to the Afrikaans for the Western Cape version (item 2 = 0.597; items 3 = 0.707; items 7 = 0.875, item 8 = 0.845 and item 22 = 0.772).

We then continued by testing if scalar invariance is supported by comparing M2: metric and M3: scalar invariance [29]. The null hypothesis being that the M2: metric and M3: scalar invariance models fit the data equally. Here the failure to reject the null hypothesis (*p* = 1) indicates that scalar invariance holds, i.e. factor loadings and the mean values across the factors are the same, supporting scalar measurement invariance (cross-cultural validity) between the Afrikaans for the Western Cape and the South African English DASH questionnaire.

## Discussion

This study is the first on structural and cross-cultural validity of the DASH questionnaire following translation and cross-cultural adaptation into a South African language [13]. In addition, novel approaches were followed during translation and cross-cultural adaptation of the Afrikaans for the Western Cape DASH questionnaire, to ensure relevance for the intended population [9–11]. Evidence of structural and cross-cultural validity is invaluable to allow clinical utility of the measure within the South African context and towards ‘validating’ novel approaches. Our sample (n = 219) is representative of the patient population typically seen in the public health sector within the South African context[4], with only 33% (n = 73) having graduated from high school and 8.2% (n = 8.2%) completing tertiary education. The inability to attain education is a contributor to unemployment [4], also reflected in the sample with almost half the participants being unemployed (46.1%, n = 101). Participants had a variety of upper limb conditions and injuries; the inclusion of distal and proximal conditions was important to validate the Afrikaans for the Western Cape DASH as an instrument that has application across the whole upper limb [16].

The decision to conduct CFA towards confirming a one-factor structure in the Afrikaans for the Western Cape DASH follows best practice in accordance with the COSMIN guidelines [25, 34], and in line with previous evidence supporting the unidimensionality of this instrument [17–23, 35, 36]. Several studies on the structural validity of the DASH questionnaire following translation and cross-cultural adaptation however did not support unidimensionality [37–46]. It is worth noting that many of these studies, did not investigate unidimensionality through CFA, but rather through EFA [37–42, 44, 46]; and in two instances IRT approaches (Rasch analysis) [45, 47] were used. In the case of the Italian DASH questionnaire, Franchignoni et al. conducted EFA, followed by CFA and Rasch analysis [43]. CFA conducted on the Afrikaans for the Western Cape DASH did not support a one-factor structure, as fit indices were below acceptable levels, adding to the growing body of evidence that questions the unidimensionality of the DASH. In fact, the evidence in support of both unidimensionality and multidimensionality is equivocal, leaving researchers with options for validating translated and cross-culturally adapted DASH questionnaires. One could proceed, as Van Eck et al. did, and explore a number of different factor structures through CFA to test hypotheses in this regard [20]. We however decided to conduct EFA to explore the number of factors in this instrument and the possibility that subscales may exist which revealed a two-factor structure. Factor analysis of the Nigerian Igbo version of the DASH questionnaire also revealed a two-factor structure [42]. This study from Nigeria is one of few (excluding the current study) reporting on language versions from the African continent and to our knowledge the only to report a two-factor model [17, 41, 42, 48, 49]. Our findings were similar to that of Ibikunle et al. (Igbo DASH) with their Factor 1 (strength based) also comprising of items 1–21 explaining 58.5% of the variance and Factor 2 (pain based) items 22–30 explaining 6.8% of the variance [42]. Others, even though there were often more than two factors identified, have also reported one major factor explaining the largest percentage of the variance [18, 39, 41, 50]. Internal consistency assessed by Chronbach’s alpha was useful to confirm the internal consistency of the two factors of the Afrikaans for the Western Cape DASH questionnaire with α ˃ 0.90 demonstrating high between-item correlation in each of the subscales (Physical function and Biopsychosocial symptoms) [14, 15]. Subsequently CFA confirmed the two-factor structure of the instrument with appropriate fit indices and acceptable levels of loading on the identified factors. To date, only three studies have conducted CFA on translations of the DASH questionnaire [20, 37, 43], the current study being the fourth to do so.

To the best of our knowledge, this is the first study conducted on the DASH questionnaire following translation and cross-cultural adaptation that evaluated cross-cultural validity (MI) through MGCFA confirming that three invariance models fit the data. Configural invariance (M1), the simplest of the MI models, does not yet consider item means but only if the same items load on the same factors in both language versions as confirmed in our sample [30, 31]. The implications of configural invariance are that across the two language versions items load on the same factors [30]. Metric invariance, still not concerned with item means, suggests that there is a similarity between the two language versions in terms of the degree to which each of the 30 items contributes to the factor(s) [30, 31]. This is achieved by constraining the factor loadings to be equivalent in both language versions [30]. During hypothesis testing (comparing M1: configural and the constraint M2: metric) metric invariance was not supported as the loadings were not found to be equal across language versions, as evident from the significant *p* value. The items that demonstrated this best for factor 1 (physical function) were items 2 (writing), 3 (turning a key), 7 (doing heavy household chores), 8 (garden or doing yard work), and factor 2 (biopsychosocial symptoms), item 22 (interference with social activities). As we assessed MI for language, taking a closer look at the translations were necessary as persons may not have understood the meaning between the two versions to be the same, resulting in factor loadings not being equivalent. No explanation can be provided for item 2 as in both language versions item 2 is one word (Afrikaans for the Western Cape: *skryf* and South African English: *write*), *skryf* being a direct translation, with no synonyms. In considering the other items it could be that during the process of translation and cross-cultural adaptation [10, 11], despite our best efforts, the meaning across language versions changed. However, when considering that the items still load on the same factors (with factor loadings of 0.597 and above), appropriately fitting M1: configural invariance, just not sufficient to support metric invariance, it may not be necessary to investigate further. Lastly, scalar invariance (M3) is concerned with mean differences in the factor(s) across the two groups (language versions) and is reflective of the fact that the mean differences between variables (factors) are the same [30, 31]. During model comparisons of M2: metric invariance and M3: scalar invariance, in testing the hypothesis to confirm scalar invariance, the constraints as applied above are retained [30]. In addition, the item means are constrained to be the same across the two language versions [30]. Scalar invariance is supported as the constraints on the item means did not significantly affect the model fit, providing evidence of cross-cultural validity (MI) between the Afrikaans for the Western Cape and South African English DASH questionnaires allowing for comparison of item means across the two language versions. It could be viewed as a limitation that we did not proceed to test residual invariance in our sample. Residual invariance tests equivalence of metric and scalar item residuals. The motivation for testing configural, metric and scalar invariance only, is based on comparison as outlined by De Vet et al. in discussing cross-cultural validity and the assessment of MI [31] as well as examples from other fields [51, 52]. Strengths of the study include that the sample size was adequate for the intended analysis and robust COSMIN methods were followed. Recommendations include evaluation of MI for other variables, such as age, sex or diagnostic grouping. Rasch analysis could also be explored towards an improved version, able to score subscales.

## Conclusion

During this study we evaluated the structural validity, internal consistency and the cross-cultural validity (MI) of the Afrikaans for the Western Cape DASH questionnaire. It has demonstrated structural validity across two factors (physical function and biopsychosocial symptoms) as well as internal consistency in both factors (subscales). Scalar invariance is supported, allowing for comparisons between the Afrikaans for the Western Cape and the South African English DASH questionnaire in research and clinical practice. Both versions are recommended for future use.

## Supplementary Information

**Additional file 1.** Construct validity of the South African English DASH questionnaire.

## Data Availability

The data that support the findings of this study are available from the corresponding author, [SdK], upon reasonable request.

## Abbreviations

PROM: Patient reported outcome measure
DASH: Disabilities of the Arm, Shoulder and Hand questionnaire
CFA: Confirmatory factor analysis
EFA: Exploratory factor analysis
MGCFA: Multiple group confirmatory factor analysis
COSMIN: The Consensus-based Standards for Selection of Health Measurement Instruments
MI: Measurement invariance
RMSEA: Root mean square error of approximation
SRMR: Standardised root mean square residual
CFI: Comparative fit index
KMO: Kaiser–Meyer–Olkin measure
