# Supplementary material for: Structural and cross-cultural validity of the Afrikaans for the Western Cape Disabilities of the Arm, Shoulder and Hand (DASH) questionnaire
Source: J Patient Rep Outcomes. 2023 Jan 11;7:1. doi: 10.1186/s41687-022-00536-w (PMC9834491; doi:10.1186/s41687-022-00536-w)
Supplement: Supplementary file 1 — Additional file 1. Construct validity of the South African English DASH questionnaire. [file 41687_2022_536_MOESM1_ESM.docx]

Supplementary Material 1

Supplementary material 1 outlines the results of confirmatory factor analysis (CFA), exploratory factor analysis (EFA) and subsequent CFA of the two-factor structure of the South African English Disabilities of the Arm, Shoulder and Hand (DASH) questionnaire on a sample of 219 participants (as outlined in the body of the manuscript).

Contents

[South African English DASH (n = 219) 1](#_Toc103248102)

[Internal Consistency 1](#_Toc103248103)

[Confirmatory Factor Analysis (n = 219) 1](#_Toc103248104)

[Exploratory Factor Analysis (n = 219) 1](#_Toc103248105)

[Eigen Values 2](#_Toc103248106)

[Factorability of the data 2](#_Toc103248107)

[Oblimin rotation and internal consistency of each subscale (two factors) 2](#_Toc103248108)

[Confirmatory Factor Analysis (two factor model) 3](#_Toc103248109)

## South African English DASH (n = 219)

### Internal Consistency

Table 1:Internal Consistency South African English DASH

| DASH | Crohnbach Alpha under the assumption of unidimentionality (95% CI) |
| --- | --- |
|  |  |
| South African English | 0.97 (0.97, 0.98) |

### Confirmatory Factor Analysis (n = 219)

Table 2: Confirmatory Factor Analysis (one factor model) – fit statistics

| Model | n | *X^2 ( df)^* | *p* | RMSEA (90% CI) | SRMR | CFI |
| --- | --- | --- | --- | --- | --- | --- |
| CFA One Factor Model (English) | 219 | 1632.04 (405) | 0.000 | 0.118 (0.112-0.124) | 0.08 | 0.988 |

RMSEA close to or less than 0.06; SRMR close to or less than 0.08; CFI close to or higher than 0.95. Unidimentionality not supported.

### Exploratory Factor Analysis (n = 219)

Exploratory Factor Analysis (EFA) with parallel analysis Scree plot:

Figure 1:Scree plot parallel analysis (South African English DASH)

Parallel analysis support two factor structure in the South African English DASH.

### Eigen Values

Table 3:Eigen Values South Africa English DASH

| English | Eigenvalue | Percentage variance | Cumulative percentage |
| --- | --- | --- | --- |
| F1 | 16.58 | 55 | 55 |
| F2 | 2.08 | 7 | 62 |

Two factors namely Physical Function and Biopsychosocial symptoms emerged with Eigen values greater than 1 explaining 55% and 7% of the variance.

### Factorability of the data

The Kaiser-Meyer-Olkin (KMO) measure of sampling adequacy value was 0.96 (above the recommended value of 0.6) and statistical significance was reached with Bartletts test of Sphericity: *X*^2^ = 5673.4, *df* = 435, p =<0.01, confirming factorability of the data.

### Oblimin rotation and internal consistency of each subscale (two factors)

Table 4:Pattern Matrix following Oblimin rotation

| DASH items | Factor 1  (Physical Function) | Factor 2 (Biopsychosocial symptoms) |
| --- | --- | --- |
| 1 | -0.73 |  |
| 2 | -0.68 |  |
| 3 | -0.76 |  |
| 4 | -0.85 |  |
| 5 | -0.7 |  |
| 6 | -0.74 |  |
| 7 | -0.81 |  |
| 8 | -0.85 |  |
| 9 | -0.77 |  |
| 10 | -0.73 |  |
| 11 | -0.7 |  |
| 12 | -0.91 |  |
| 13 | -0.79 |  |
| 14 | -0.8 |  |
| 15 | -0.81 |  |
| 16 | -0.81 |  |
| 17 | -0.8 |  |
| 18 | -0.77 |  |
| 19 | -0.8 |  |
| 20 | -0.74 |  |
| 21 | -0.72 |  |
| 22 |  | -0.57 |
| 23 | **-0.4** | **-0.45** |
| 24 |  | -0.96 |
| 25 |  | -0.81 |
| 26 |  | -0.79 |
| 27 |  | -0.82 |
| 28 |  | -0.65 |
| 29 |  | -0.76 |
| 30 |  | -0.57 |
| Eigenvalue | 16.58 | 2.08 |
| Percentage variance | 55% | 7% |
| Cumulative percentage | 55% | 62% |
| Cronbach’s Alpha (95% CI) | 0.97(0.96, 0.97) | 0.92(0.90, 0.94) |

### Confirmatory Factor Analysis (two factor model)

Table 5:Confirmatory Factor Analysis (two factor model) – fit statistics

| Model | n | *X^2 ( df)^* | *p* | RMSEA (90% CI) | SRMR | CFI |
| --- | --- | --- | --- | --- | --- | --- |
| CFA Two Factor Model (South African English DASH) | 219 | 760.41 (404) | 0.000 | 0.064 (0.057-0.071) | 0.05 | 0.996 |

RMSEA close to or less than 0.06; SRMR close to or less than 0.08; CFI close to or higher than 0.95, fit statistics confirms two factor structure of the South African English DASH.
